# Enhancement in Seed Priming-Induced Starch Degradation of Rice Seed Under Chilling Stress via GA-Mediated α-Amylase Expression

**DOI:** 10.1186/s12284-022-00567-3

**Published:** 2022-03-27

**Authors:** Lixiao Nie, Shaokun Song, Qi Yin, Tingcheng Zhao, Hongyan Liu, Aibin He, Weiqin Wang

**Affiliations:** 1 Hainan Key Laboratory for Sustainable Utilization of Tropical Bioresource, College of Tropical Crops, Hainan University, Haikou, China; 2 College of Agronomy, Hunan Agricultural University, Changsha, China

**Keywords:** Direct-seeded rice, Chilling stress, Seed priming, Starch degradation, Hormonal metabolism

## Abstract

Chilling stress is the major abiotic stress that severely limited the seedling establishment of direct-seeded rice in temperate and sub-tropical rice production regions. While seed priming is an efficient pre-sowing seed treatment in enhancing crop establishment under abiotic stress. Our previous research has identified two seed priming treatments, selenium priming (Se) and salicylic priming (SA) that effectively improved the seed germination and seedling growth of rice under chilling stress. To further explore how seed priming enhance the starch degradation of rice seeds under chilling stress, the present study evaluated the effects of Se and SA priming on germination and seedling growth, α-amylase activity, total soluble sugar content, hormone content and associated gene relative expression under chilling stress. The results showed that both Se and SA priming significantly increased the seed germination and seedling growth attributes, and enhanced the starch degradation ability by increasing α-amylase activity and total soluble sugar content under chilling stress. Meanwhile, seed priming increased the transcription level of *OsRamy1A*, *OsRamy3B* that regulated by GA, and increased the transcription level of *OsRamy3E* that regulated by sugar signals. Furthermore, seed priming significantly improved the GA_3_ contents in rice seeds by up-regulating the expression of *OsGA3ox1* and *OsGA20ox1*, and decreased the ABA content and the expression of *OsNCED1,* indicating that the improved starch degradation ability in primed rice seeds under chilling stress might be attributed to the increased GA_3_ and decreased ABA levels in primed rice seeds, which induced the expression of GA-mediated α-amylase. However, studies to explore how seed priming mediate hormonal metabolism and the expression of *OsRamy3E* are desperately needed.

**Supplementary Information:**

The online version contains supplementary material available at 10.1186/s12284-022-00567-3.

## Introduction

Direct-seeded rice (DSR), a simplified and efficient rice establishment method that direct sowing the rice seeds into field, was suggested to be an alternative strategy to replace the traditional transplanting because of its benefits on saving labor and resources input, reducing greenhouse gas emission and increasing water productivity (Wang et al. 2017). Nevertheless, in several sub-tropical rice production regions, the DSR was easily suffered from chilling stress after sowing, resulting in poor crop establishment and unstable yield, which largely limited the promotion and extension of DSR (Zhang and Zhu 2020). Although the threshold temperature for rice seed germination is 10–12 °C, the seedling establishment was significantly inhibited when the temperature was lower than 20 °C (Ueno and Miyoshi 2005). Chilling stress would depress several metabolic events during rice seed germination including starch degradation (Oliver et al. 2007; Ruelland et al. 2009; Lee et al. 2014). In rice seeds, the starch degradation process is the fundamental metabolic event to guarantee energy supply and respiration during germination. The starch degradation during rice seed germination is regulated by α-amylase, β-amylase, starch debranching enzyme and maltase. While α-amylase is the most important enzyme and its activity was highly associated with germination percentage and seedling growth attributes of rice (Williams and Peterson 1973; Krishnasamy and Seshu 1989; Cui et al. 2002; Mahender et al. 2015). Nevertheless, the α-amylase was extremely sensitive to low temperature (Shaw and Lee 1984; Karrer and Rodriguez 1992; Umemoto et al. 1995). The decreased α-amylase activity under chilling stress reduced the total soluble sugar content and respiration rate in rice seeds and seedlings, thus resulted in poor establishment of DSR under chilling stress (Wang et al. 2016). Therefore, effective strategies to enhance the starch degradation ability and germination performance of DSR under chilling stress are desperately needed.

Seed priming, which refers to one of the pre-sowing seed treatments that controls seed hydration process to trigger several metabolic processes during early phase of germination before radicle protrusion (Hussain et al. 2016), has been proved to be an effective, practical and facile technique to enhance seed germination, seedling growth and crop yield particularly under unfavorable conditions (Paparella et al. 2015; Jisha and Puthur 2016). Previously, the ability of seed priming to enhance chilling tolerance during crop establishment has been reported in carrot, maize, tobacco, chickpea and sunflower (Moosavi et al. 2009; Patade et al. 2009; Balestrazzi et al. 2011; Khaliq et al. 2015). Our previous studies also identified two seed priming treatments, viz. Selenium priming (Se) and Salicylic acid priming (SA), were effective in enhancing the seed germination and seedling growth attributes of rice under chilling stress (Wang et al. 2016a; Hussain et al. 2016; Nie et al. 2020). The mechanisms contributing to the enhanced stress tolerance in primed seeds includes improved respiratory metabolism (Corbineau et al. 2000; Weitbrecht et al. 2011; Nie et al. 2020), stronger activity of reactive oxygen species scavenging system (Maia et al. 2011; Chen and Arora 2013) and increased membrane integrity (Moosavi et al. 2009; Patade et al. 2009). In particular, several previous study and our researches have observed that seed priming enhanced the starch degradation process under chilling stress by increasing α-amylase activity and total soluble sugar content (Hussain et al. 2016; Wang et al. 2016a; Wang et al. 2016b; Zheng et al. 2016). And such improvement was strongly associated with better germination performance of primed rice seeds under chilling stress. Nevertheless, the mechanism underlying how seed priming increased α-amylase activity under chilling stress remained to be unknown.

In rice seeds, the α-amylase is encoded by a multigene family of at least 10 genes, but only 3 α-amylase isoforms, A, B and E, were identified in germinating rice seeds (Huang et al. 2000). These 3 α-amylase isoforms were encoded by 4 major α-amylase genes, *OsRamy1A*, *OsRamy3B*, *OsRamy3D* and *OsRamy3E.* Of which *OsRamy1A* was the most abundant amylase gene during seed germination which encodes isoforms A and B, and its transcription is regulated by gibberellin acid (GA) and abscisic acid (ABA) (Lee et al. 2014). Previously, the regulating effect of GA and ABA on the induction of α-amylase during seed germination has been observed by several researches (Gubler et al. 1995; Miyuki et al. 2004; Park et al. 2010). Gubler et al. (1995) firstly identified a gibberellin (GA) response complex GAMYB in the aleurone layers of barley. The GAMYB protein induced the expression of α-amylase and some other GA-inducible genes by interacting directly with the GA-responsive cis-acting elements (GAREs) of these genes in aleurone tissue (Gubler et al. 1995). Meanwhile, the research of Ritchie and Gilroy (1998) suggested that the accumulation of ABA in seed tissues depressed the expression of GAMYB protein, thus decreased the production of α-amylase that solely regulated by GA signals. In addition to hormonal metabolism, the expression of α-amylase was also regulated by sugar signals. It has been suggested that the increase of sugar content in plant tissues would down-regulate the transcription level of *Ramy3* family gene. (Mitsui et al. 1999; Lu et al. 2009; Park et al. 2010). But such effect might be inverted by the increase of GA content. In addition, it has been found that the promoter sequence of *OsRamy3D* and *OsRamy3E* did not contain GAREs, suggesting that the transcription of these *Ramy3* genes were mainly regulated by sugar signals rather than GAs (Karrer and Rodriguez 1992, Thomas and Rodriguez 1994).

Based on the previous studies, it can be speculated that the enhanced starch degradation ability in primed rice seeds under chilling stress might be attributed to the increases of the GA-mediated α-amylase gene expression which are regulated by the GA and ABA biosynthesis and degradation. Meanwhile, seed priming might also affect the transcription level of *Ramy3* family that mediated by sugar signals. Nevertheless, further studies are needed to explore the potential pathway that regulates the starch metabolism in primed rice seeds under chilling stress. Therefore, the objectives of the present study were to evaluate the effects of seed priming on germination performance and starch degradation ability of rice seeds under chilling stress, and to explore the potential mechanisms underlying the priming induced effects on starch degradation of rice seeds under chilling stress regarding hormone content and relative gene transcription.

## Materials and Methods

### Seed Sources

Seeds of two early rice varieties that were widely used in central China, Ezao18 (EZ18) and Liangyou287 (LY287), were obtained from the Crop Physiology and Production Center, Huazhong Agricultural University, Wuhan, China. Both cultivars have initial germination of > 95% at 25 °C. The initial seed moisture content was < 10.0% (on a dry weight basis).

### Seed Priming Treatment

Previously, we compared the effectiveness of different seed priming techniques including hydropriming (distilled water), osmo-priming (CaCl_2_: 100 mg L^−1^ calcium chloride), redox priming (H_2_O_2_: 50 µM hydrogen peroxide), nutri-priming (Se: 50 µM selenium) and hormonal priming (SA: 100 mg L^−1^ salicylic acid) under chilling stress conditions at different set of temperatures and cultivars under controlled growth chamber conditions. We found that Se and SA priming treatments were more effective in enhancing chilling tolerance than all other priming treatments. Therefore, Se and SA priming were selected as the priming treatments in this present study. The procedures for seed priming were as follows: The seeds were sterilized in 10% hydrogen peroxide solution for 10 min. After then, the seeds were washed with distilled water and then transferred to the priming solution (Se: 50 µM selenium; SA: 100 mg/L salicylic acid). The priming condition was set as 25 °C for 24 h. The ratio between seed weight to the volume of priming solution was set as 1:5. After priming, the primed seeds were carefully washed and then transferred to an oven and oven-dried at moderate temperature (25 °C) until the seeds moisture contents were below 10%. The primed seeds were stored at 4 °C and vacuum condition for no longer than 15 days before experiments.

### Experimentation

The experiments were conducted in two growth chambers and were arranged in complete randomized design. The treatments were No-priming-Normal Temperature (NT); No priming-Chilling Stress (CK); Se priming-Chilling stress (Se) and SA priming-Chilling stress (SA). Previously, several studies have shown that the rice seed germination and seedling growth would be severely inhibited when germinated under the temperature of 16–20 °C (Yoshida 1981; Sipaseuth et al. 2007; Wang et al. 2016a; Nie et al. 2020). Therefore, the temperature for chilling stress was set as 16 °C, and the temperature for normal temperature treatment was set as 25 °C. The chilling stress and normal temperature treatment was conducted in two growth chambers with the 12-h light period and air humidity of 60% ± 5%, respectively.

Fifty seeds with similar size were germinated on two layers of filter paper in petri dishes (14.5 cm) and were arranged in 5 rows (10 seeds for each row). After adding 20 ml distilled water, the petri dishes were then transferred to growth chamber. All the treatments were replicated for 12 times. Three replications were used for observation of germination dynamics and seedling growth attributes. Another three replications were used to determine α-amylase activity and total soluble sugar content. Another three replications were used for determination of gene relative expression levels. While the last three replications were used for GA_3_ and ABA content assay. When sampling, 10 seeds from one of the 5 rows were randomly selected for further determination and both germinated and ungerminated seeds were selected.

### Observations

#### Germination and Seedling Attributes

The seed germination percentage was measured daily according to the procedure reported by AOSA (1990). Seed was considered to be germinated when radicle length exceeded 2 mm. At 10 days after sowing, 10 rice seedlings from each petri dishes were sampled, and after measuring the root length and shoot length, the roots and shoots were then separated to measure the root and shoot fresh weight.

#### α-Amylase Activity

For determination of α-amylase activity, 0.3 g rice seeds were sampled at 3, 6 and 9 DAS. After de-hulling, the seeds were homogenized and rinsed with 8 ml ice-cold Na-phosphate buffer (pH 7.0, 0.1 M). After centrifuging at 12,000 g for 20 min, the supernatant was collected as a crude extract. The DNS method was used to determine the α-amylase activity (Bernfeld 1955).

#### Total Soluble Sugar Content

The determination of the total soluble sugar contents was according to the methods of Zheng et al. (2016), 0.3 g frozen seedling samples of each treatment were ground and mixed with 50 ml distilled water. Then the mixture was filtered using Whatman No. 42 filter paper. The total soluble sugar contents in rice seedlings were evaluated by the phenol sulfuric method (Dubois et al. 1956).

#### RNA Extraction, cDNA Synthesis and Quantitative Real-Time PCR

Total RNA was extracted from approximately 150 mg of the seed embryos at 3, 6 and 9 DAS using the RNAprep PurePlant kit (Tiangen Biotech, Beijing, China). Total RNA was used to synthesize cDNA with random oligonucleotides using a PrimeScript™ RT reagent Kit with gDNA Eraser (Takara, www.takara-bio.com). Quantitative RT-PCR was performed using an QuantStudio™ 6 Flex fast Real-Time PCR System (Applied Biosystems) with FastStart Universal SYBR Green Master (Roche Molecular Systems, lifescience.roche.com). A rice ACTIN gene was used as an internal control. The relative quantification of the transcript levels was performed using the comparative Ct method (Livak and Schmittgen 2001). The primers used in the present study were shown in Additional file 1: Table S1.

#### *GA*_*3*_* and ABA Content*

The GA_3_ and ABA were extracted and purified by the method of Yang et al. (2016) with slight modifications. A 200 mg of de-hulled rice seeds was homogenized with liquid nitrogen and then extracted in 3 ml 80% methanol at 4 °C for 24 h and then centrifuged at 12,000 rpm for 15 min at 4 °C. The supernatant was passed through C18.

Sep-Pak cartridges (Waters Corp., Milford, MA, USA), and the phytohormone fraction was eluted with 10 mL of methanol and 10 mL of ether. The eluate was dried under pure N_2_ at 20 °C and then resuspended in 100 μL of 100% methanol. 2 μL of each sample (each biological replication was replicated for three times) was injected into a 8050 LCMS system (Shimadzu corporation), and the eluted ions were monitored by MRM.

### Statistics Analysis

All data from present experiment were expressed as the mean value of three biological replications. Statistix 11.0 was used to analyze the data using least significant difference (LSD) test at 0.05 probability level. The figure illustration was performed using SigmaPlot 12.5.

## Results

### Seed Germination and Seedling Growth

**Fig. 1:**
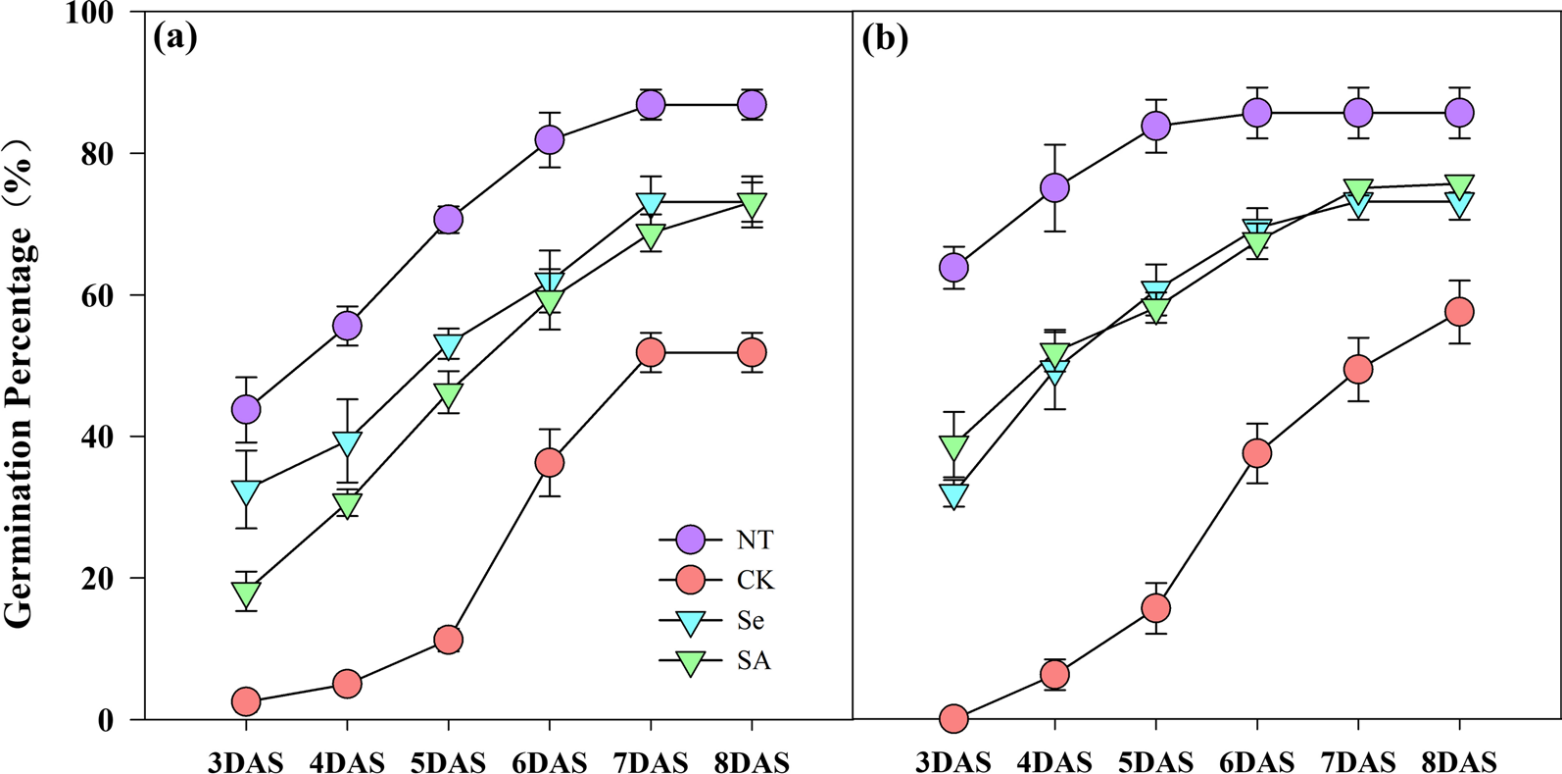
Germination dynamics of primed and non-primed rice seeds under control temperature and chilling stress. **a** EZ18. **b** LY287. DAS, days after sowing; EZ18, ezao18; LY287, liangyou287; NT, no priming-normal temperature control; CK, no priming-chilling stress; Se, Selenium priming-chilling stress; SA, Salicylic priming-chilling stress. Seed germination data was recorded from 3DAS till constant at 8 DAS. Error bars indicate standard error (n = 3)

Chilling stress severely reduced the germination percentage of rice seeds (Fig. 1). When compared with normal temperature control, the germination percentage in EZ18 and LY287 was decreased by 44.4% and 37.8%, respectively, under chilling stress. Meanwhile, both Se and SA priming increased the germination percentage of rice seeds under chilling stress. In EZ18, the germination percentage of Se-primed and SA-primed rice seeds was increased by 41.1% and 41.3%, respectively as compared with un-primed control. Similarly, the increases of 27.1% and 31.4% on germination percentage was observed in Se-primed and SA-primed rice seeds in LY287, respectively as compared with un-primed control.

Chilling stress inhibited the growth of rice seedlings, but such inhibition was partially relieved by seed priming treatments (Table 1). Averaging across priming treatments and cultivars, the root length, shoot length, root fresh weight and shoot fresh weight of primed rice seedlings was increased by 67.3%, 76.3%, 30.7% and 78.0%, respectively as compared with non-primed control under chilling stress. The variance in seed germination and seedling growth attributes between the two varieties were similar in response to chilling stress and priming treatments, and no significant interaction was found between variety and seed treatments.

**Table 1:** The root length, shoot length, root fresh weight, and shoot fresh weight of Se-primed, SA-primed, and unprimed seeds of rice under control temperature and chilling stress

Variety	Treatment	Root length (cm)	Shoot length (cm)	Root fresh weight (mg seedling^−1^)	Shoot fresh weight (mg seedling^−1^)
EZ18	NT	5.1 a	3.9 a	13.46 a	21.84 a
	CK	2.3 c	1.9 c	6.59 c	8.62 c
	Se	3.9 b	3.4 ab	8.12 bc	14.28 b
	SA	4.0 b	3.2 b	9.34 b	14.40 b
LY287	NT	5.2 a	3.9 a	12.56 a	21.41 a
	CK	2.4 c	1.9 c	6.63 b	8.78 c
	Se	3.8 b	3.3 b	8.06 b	17.06 b
	SA	3.7 b	3.5 ab	8.97 b	17.15 b

Within a column for each variety, different lowercase letters denote statistical differences at the 5% level according to LSD test. EZ18, ezao18; LY287, liangyou287; NT, no priming-normal temperature control; CK, no priming-chilling stress; Se, Selenium priming-chilling stress; SA, Salicylic priming-chilling stress

### α-Amylase Activity and Soluble Sugar Content

**Fig. 2:**
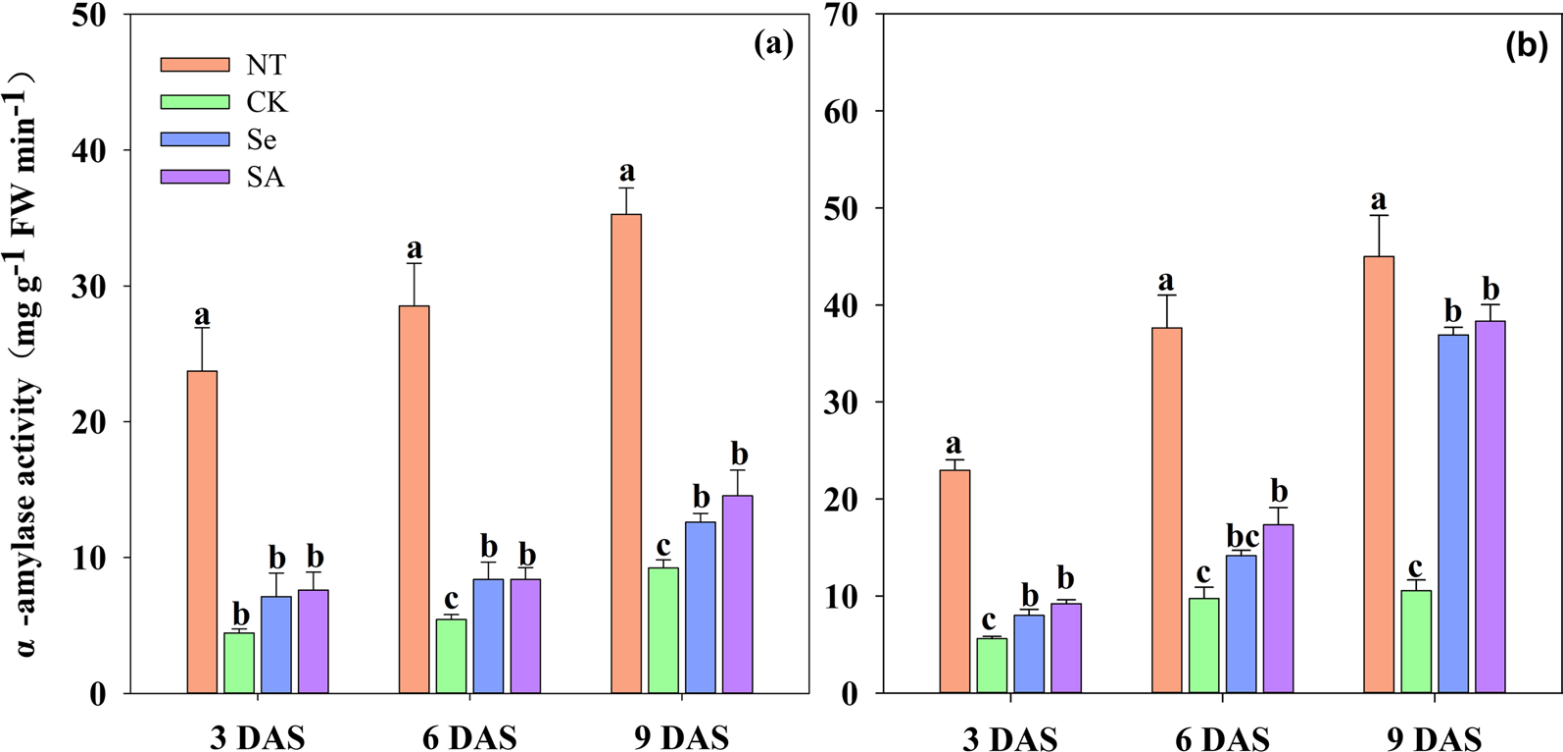
Changes in α-amylase activity of primed and non-primed rice seeds under control temperature and chilling stress. **a** EZ18. **b** LY287. DAS, days after sowing; EZ18, ezao18; LY287, liangyou287; NT, no priming-normal temperature control; CK, no priming-chilling stress; Se, Selenium priming-chilling stress; SA, Salicylic priming-chilling stress. Error bars indicate standard error (n = 3). Different lowercase letters denote statistical differences at the 5% level according to LSD test among four treatments within the same DAS of the same variety

**Fig. 3:**
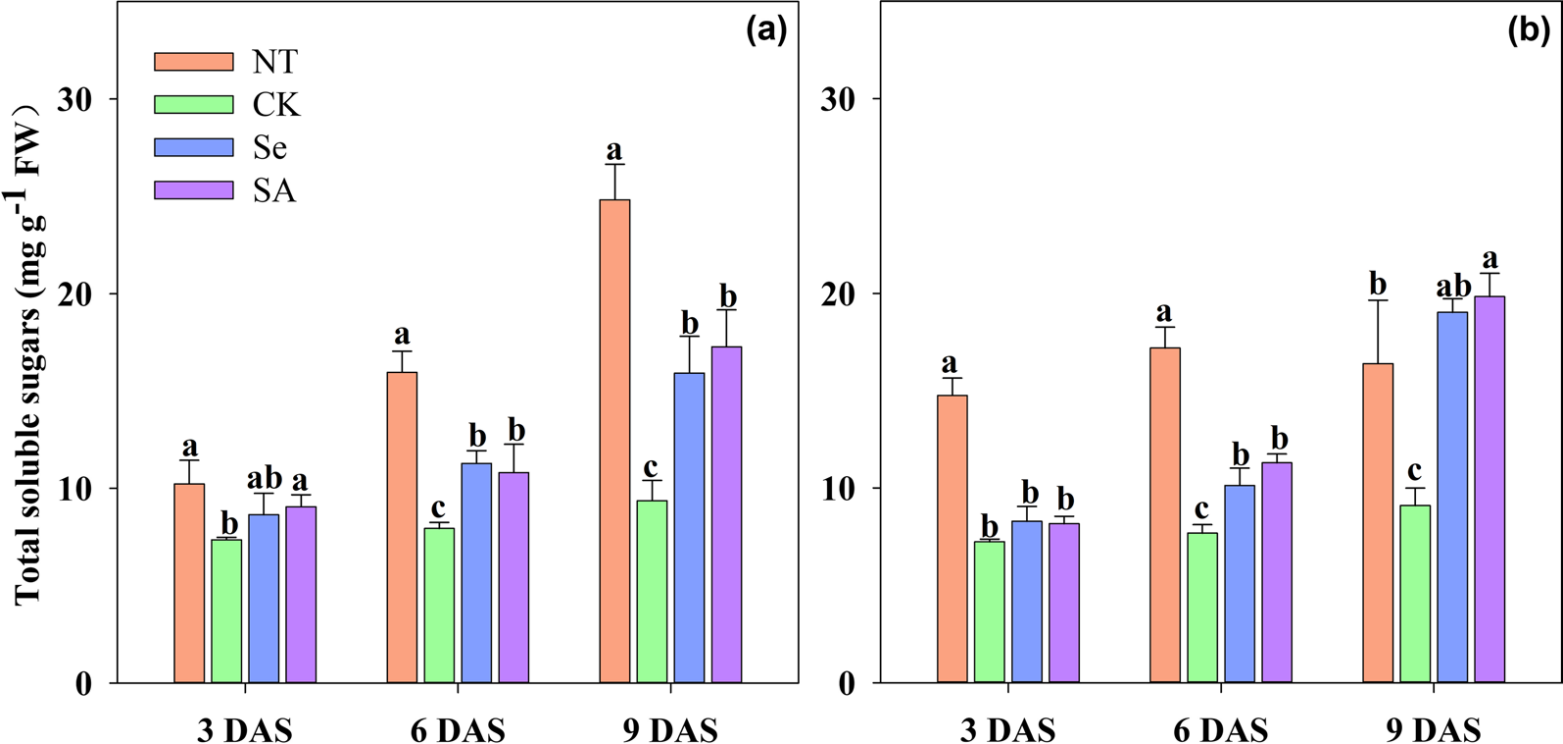
Changes in total soluble sugar contents of primed and non-primed rice seeds under control temperature and chilling stress. **a** EZ18. **b** LY287. DAS, days after sowing; EZ18, ezao18; LY287, liangyou287; NT, no priming-normal temperature control; CK, no priming-chilling stress; Se, Selenium priming-chilling stress; SA, Salicylic priming-chilling stress. Error bars indicate standard error (n = 3). Different lowercase letters denote statistical differences at the 5% level according to LSD test among four treatments within the same DAS of the same variety

Chilling stress severely limited the α-amylase activity and decreased the total soluble sugar contents in rice seeds and seedlings (Figs. 2, 3). When compared with NT, the α-amylase activity of CK treatment was decreased by 78.4%, 77.0% and 75.3% at 3, 6 and 9 DAS, respectively. Meanwhile, chilling stress decreased the total soluble sugar content in rice seeds and seedlings by 41.6–55.2% respectively, averaged across cultivars.

Both Se and SA priming accelerated the process of starch metabolism under chilling stress (Figs. 2, 3) At 3 DAS, the α-amylase activity in primed rice seeds and seedlings was significantly improved as compared with that of CK. And such improvement was maintained from 3 to 9 DAS. Averaging across sampling days and cultivars, the α-amylase activity in Se and SA primed rice seeds/ seedlings was increased by 150.1% and 167.3%, respectively as compared with CK. For total soluble sugar content, no significant variance was observed between primed and non-primed rice seeds at 3 DAS in response to chilling stress. However, a progressively increase in soluble sugar content was observed in primed rice seeds and seedlings from 6 to 9 DAS. Averaging across priming treatments and varieties, seed priming increased the soluble sugar content in rice seeds by 21.8% and 62.7%, respectively at 6 DAS and 9 DAS, as compared with that in non-primed control. Among varieties, both EZ18 and LY287 showed similar responses to chilling stress and seed priming treatment from 3 to 9 DAS.

### Relative Expression of α-Amylase Genes

**Fig. 4:**
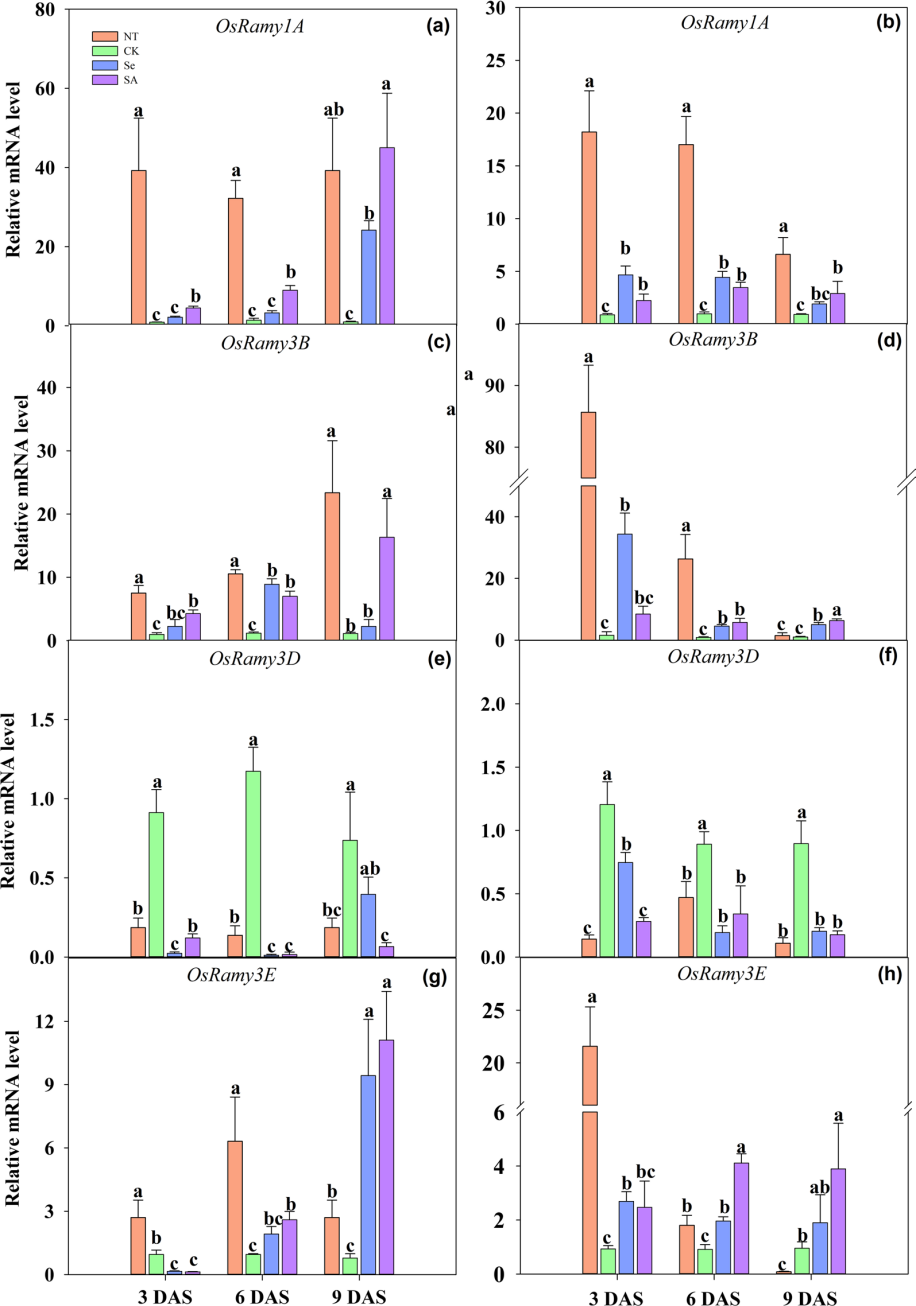
Relative expression levels of α-amylase genes of primed and non-primed rice seeds under control temperature and chilling stress. **a, c, e, g** EZ18; **b, d, f, h** LY287; **a, b** *OsRamy1A*; **c, d** *OsRamy3B*; **e, f** *OsRamy3D*; **g, h** *OsRamy3E*; DAS, days after sowing; EZ18, ezao18; LY287, liangyou287; NT, no priming-normal temperature control; CK, no priming-chilling stress; Se, Selenium priming-chilling stress; SA, Salicylic priming-chilling stress. Error bars indicate standard error (n = 3). Different lowercase letters denote statistical differences at the 5% level according to LSD test among four treatments within the same DAS of the same variety

The relative expression of different α-amylase genes responding to chilling stress and seed priming was shown in Fig. 4. For non-primed rice seeds and seedlings, the chilling stress down-regulated the relative expression of *OsRamy1A*, *OsRamy3B* and *OsRamy3E*, but increased the expression of *OsRamy3D,* as compared with normal temperature control (Fig. 4). For priming treatment, both Se and SA priming significantly up-regulated the expression of *OsRamy1A* and *OsRamy3B* which were regulated by GA. (Fig. 4). At 3 DAS, the relative expression of *OsRamy1A* and *OsRamy3B* gene in primed rice seeds and seedlings was increased by 3.7 and 15.0-fold respectively, and such improvement was maintained from 3 to 9 DAS. Averaging across cultivars and sampling days, the relative expression of *OsRamy1A* and *OsRamy3B* was up-regulated by 6.7 and 8.5 folds by Se priming and 11.0 and 7.1 folds by SA priming, respectively.

The expression of *OsRamy3D* and *OsRamy3E* was regulated by the sugar concentration in plant tissues. In the present study, significant variances on relative expression level between these two genes in response to priming treatments were observed (Fig. 4). Seed priming inhibited the expression level of *OsRamy3D* gene, which was decreased by 63.6%, 90.1% and 63.3% under Se priming, and was decreased by 81.0%, 82.7% and 85.1% under SA priming at 3, 6 and 9 DAS, respectively as compared with non-primed control. In contrary, both Se and SA priming enhanced the expression of *OsRamy3E*. When averaging across cultivars and sampling days, the relative expression level of *OsRamy3E* was increased by 3.3 and 4.4 folds in Se primed and SA primed rice seedlings respectively as compared with non-primed control.

### GA_3_ and ABA Content

**Fig. 5:**
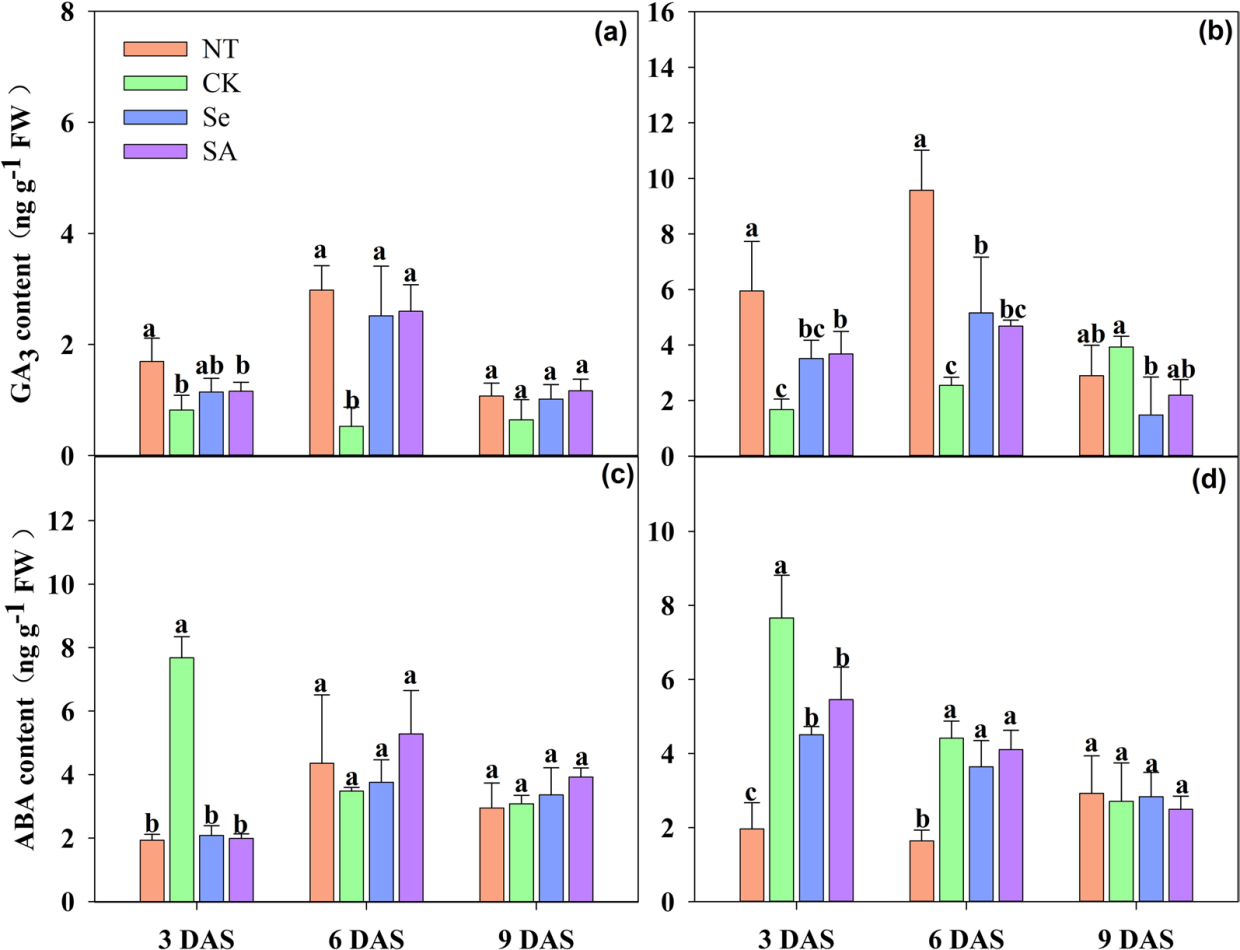
GA_3_ and ABA content of primed and non-primed rice seeds under control temperature and chilling stress. **a, c** EZ18; **b, d** LY287; **a, b** GA_3_ content; **c, d** ABA content DAS, days after sowing; EZ18, ezao18; LY287, liangyou287; NT, no priming-normal temperature control; CK, no priming-chilling stress; Se, Selenium priming-chilling stress; SA, Salicylic priming-chilling stress. GA, gibberellin acid; ABA, abscisic acid. Error bars indicate standard error (n = 3). Different lowercase letters denote statistical differences at the 5% level according to LSD test among four treatments within the same DAS of the same variety

Chilling stress inhibited the GA_3_ accumulation in rice seeds during germination, while seed priming alleviated the inhibition effect and promoted GA_3_ accumulation to some extent as compared with non-primed control (Fig. 5). When compared with normal temperature control, chilling stress decreased the GA_3_ content in rice seeds by 67.4% and 75.5%, respectively at 3 DAS and 6 DAS. Meanwhile, the GA_3_ content in Se primed and SA primed rice seeds under chilling stress was increased by 86.8% and 93.8% respectively at early germination stage (3 DAS). The GA_3_ contents in primed rice seeds continuously increased with seed germination and reached the highest value at 6 DAS. However, no difference was found in GA_3_ content between primed and non-primed rice seeds at 9 DAS.

The variances in ABA content between primed and non-primed rice seeds under chilling stress were shown in Fig. 5. Chilling stress significantly accelerated the accumulation of ABA in non-primed rice seeds at early germination stage (3 DAS), as the ABA content of that was increased by 293.4% as compared with normal temperature control. Meanwhile, the ABA content in Se primed and SA primed rice seeds was decreased by 57.0% and 51.5% respectively when compared with non-primed control, averaging across cultivars. Nevertheless, the ABA content in rice seeds under chilling stress was decreased rapidly from 6 to 9 DAS and did no show significant difference to that germinated under normal temperature. Furthermore, no variance on ABA content was observed between primed and non-primed rice seeds during 6–9 DAS. In addition, although the GA_3_ and ABA content between the two varieties showed a slight difference, no significant variance (P > 0.05) was observed between the two varieties in response to different treatments.

### Relative Expression of GA_3_ and ABA Biosynthesis and Degradation Genes

**Fig. 6:**
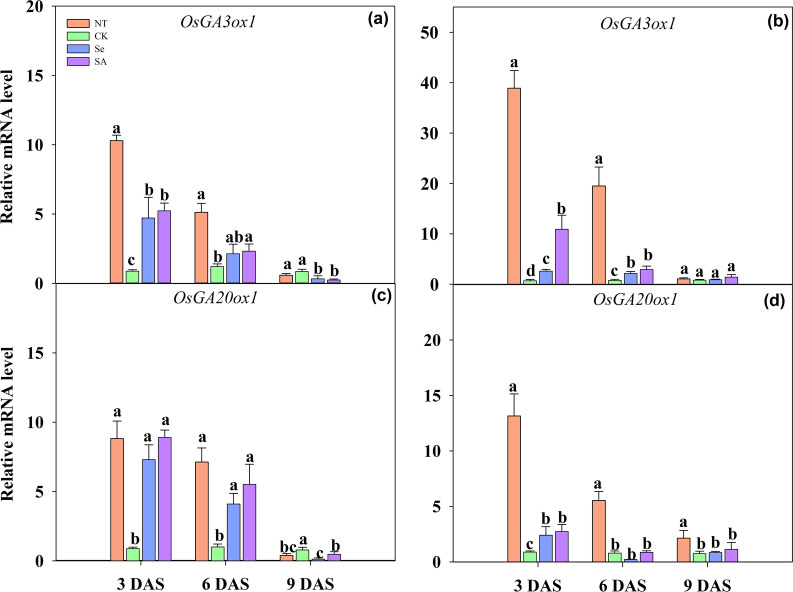
Relative expression levels of GA biosynthesis genes of primed and non-primed rice seeds under control temperature and chilling stress. **a, c** EZ18; **b, d** LY287; **a, b** *OsGA3ox1*; **c, d** *OsGA20ox1*. DAS, days after sowing; EZ18, ezao18; LY287, liangyou287; NT, no priming-normal temperature control; CK, no priming-chilling stress; Se, Selenium priming-chilling stress; SA, Salicylic priming-chilling stress. Error bars indicate standard error (n = 3). Different lowercase letters denote statistical differences at the 5% level according to LSD test among four treatments within the same DAS of the same variety

The expression of GA biosynthetic genes was shown in Fig. 6. Under chilling stress, the expression of *OsGA3ox1* and *OsGA20ox1* in non-primed rice seeds was significantly inhibited, as the relative expression level of *OsGA3ox1* and *OsGA20ox1* in non-primed rice seeds was decreased by 81.8% and 82.9% at 3 DAS, and was decreased by 76.7% and 55.3% at 6 DAS, respectively as compared with normal temperature control, averaged across cultivars. However, such inhibition that induced by chilling stress can be partially relieved by seed priming treatment (Fig. 6). Comparing with non-primed control, Se priming up-regulated the expression level of *OsGA3ox1* by 4.6 and 2.2 folds at 3 DAS and 6 DAS respectively. Similarly, SA priming increased the *OsGA3ox1* expression by 10.2 and 2.6 folds at 3 DAS and 6 DAS respectively. The expression pattern of *OsGA20ox1* responding to priming treatment was similar to that of *OsGA3ox1*, as 2.4–5.5 folds increases in Se primed seeds, and 3.6–6.6 folds increases in SA primed seeds was observed when compared with non-primed control. Nevertheless, the expression level of *OsGA3ox1* and *OsGA20ox1* in primed rice seeds was similar or even lower than that of non-primed rice seeds at 9 DAS. Besides, the expression level of *OsGA*_*20*_*ox1* in SA-primed rice seeds in LY287 at 6 DAS did not show variance to that of non-primed rice seeds.

**Fig. 7:**
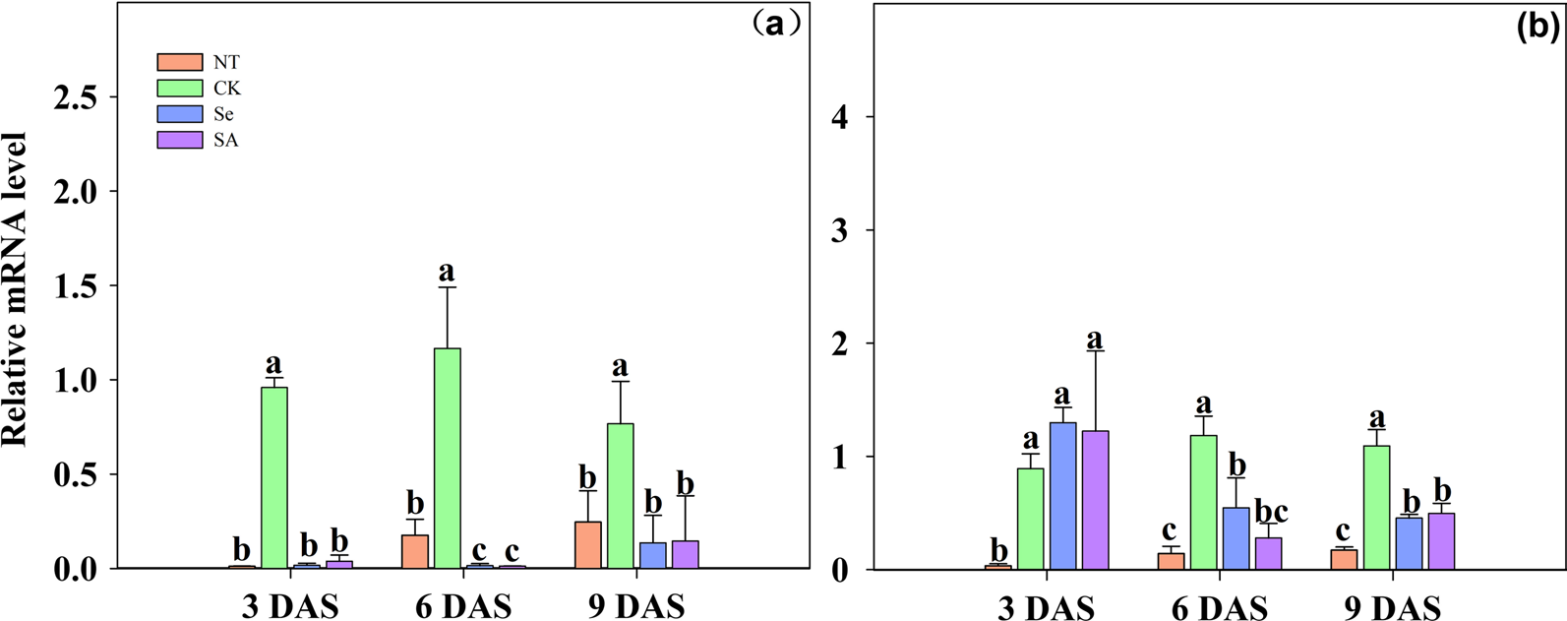
Relative expression levels of *OsNCED1* of primed and non-primed rice seeds under control temperature and chilling stress. **a** EZ18; **b** LY287. DAS, days after sowing; EZ18, ezao18; LY287, liangyou287; NT, no priming-normal temperature control; CK, no priming-chilling stress; Se, Selenium priming-chilling stress; SA, Salicylic priming-chilling stress. Error bars indicate standard error (n = 3). Different lowercase letters denote statistical differences at the 5% level according to LSD test among four treatments within the same DAS of the same variety

Chilling stress accelerated the expression of ABA biosynthetic gene *OsNCED1 *(Fig. 7)*,* while seed priming partially reduced its expression*.* Averaging across cultivars, the expression level of *OsNCED1* in non-primed rice seeds that growth under chilling stress was increased by 116.3%, 69.7% and 44.4% respectively at 3, 6 and 9 DAS, as compared with normal temperature control. In addition, a slight variance in *OsNCED1* expression level between the two varieties was observed. In EZ18, the *OsNCED1* expression was pronouncedly decreased by 87.3% in primed rice seeds from 3–9 DAS, averaged across priming treatments. In LY287, although the expression level of *OsNCED1* in primed rice seeds did not vary to that of non-primed one at 3 DAS, a significant reduction of *OsNCED1* expression was induced by both priming treatments at 6 DAS and 9 DAS.

## Discussion

Chilling stress is one of the major abiotic stresses which severely limited the germination and early seedling growth of rice. In the present study, the negative effects of chilling stress during rice seeds germination were partially relieved by seed priming treatments (Fig. 1, Table 1). Moreover, both Se and SA priming significantly enhanced the starch metabolism under chilling stress via improved α-amylase activity and total soluble sugar contents (Figs. 2, 3). And such benefits have also been widely reported in maize, tomato, onion, wheat and sorghum (Smith and Cobb 1992; Mudgett et al. 1997; Lin and Sung 2001; Rashid et al. 2004; Chen and Arora 2013). Previously, our researches have suggested that the improved α-amylase activity was highly correlated with high seed germination percentage, better seedling growth and stress tolerance during the establishment stage (Wang et al. 2016a; Wang et al. 2016b; Hussain et al. 2016). However, the metabolic pathways controlling the expression of α-amylase in primed rice seeds and seedlings has rarely been explored. The synthesis of α-amylase was a complicated physio-biochemical process which was controlled by several events including hormonal and sugar metabolism (Loreti et al. 2003). The present study found that seed priming up-regulated the expression level of *OsRamy1A*, *OsRamy3B* under chilling stress (Fig. 4). It has been proved that the expression of *OsRamy1A* was highly induced by GA, but was depressed by ABA (Mitsui and Itoh 1999; Miyuki et al. 2004; Zhao et al. 2018). While the expression of *OsRamy3B* was regulated by the soluble sugar levels in plant tissues. In addition, the promoter sequence of *OsRamy3B* contained gibberellic acids response element (GAREs) (Hwang et al. 1999; Loreti et al. 2003), which suggested that the expression of *OsRamy3B* can be also regulated by the GA signals. The results of GA and ABA content further proved that seed priming significantly increased the GA_3_ contents and decreased the ABA contents in rice seeds and seedlings under chilling stress. Therefore, it can be deduced that the increased α-amylase activity in primed rice seeds might be attributed to the changes in GA and ABA content, which promoted the expression of *OsRamy1A* and *OsRamy3B*. Several previous researches have also found that seed priming accelerated the degradation of seed reserves via regulating hormone levels in seed (Argyris et al. 2008; Shigeo et al. 2008).

To further explore how seed priming increased the GA_3_ content and inhibited the accumulation of ABA. The present study found that both Se and SA priming significantly up-regulated the expression level of *OsGA*_*3ox1*_ and *OsGA*_*20*_*ox1*, which encoded the key enzymes involved in the GA_3_ biosynthesis pathways. Previously, the positive effect of seed priming on enhancing GA synthesis in plants has been observed by several researches. Nakaune et al. (2012) indicated that seed priming promoted the expression of GA_4_ synthesis genes in tomato seeds. While the research of Li et al. (2017) found that the expression levels of *GA20ox1* and *GA3ox2* in primed maize seeds was highly up-regulated under chilling stress. In lettuce seeds, the *LsGA3ox1* was also up-regulated responding to seed priming (Argyris et al. 2008). For ABA metabolism, NCED was the key enzymes involving in ABA biosynthesis pathways, which was highly induced in plant that suffered abiotic stresses (Tan et al. 2003; Ye 2011). Our study found that the expression level of *OsNCED1* was significantly decreased in primed rice seeds as compared with non-primed ones under chilling stress, implying that the decreased ABA content in primed rice seeds might be attributed to the depressed activity of ABA biosynthesis pathways. Meanwhile, the ABA levels in plant tissues can also be determined by ABA degradation metabolism (Yang and Choi 2006, Saika et al. 2007). Nevertheless, our study found that the expression level of *OsABA8ox1* in primed rice seeds, of which encoded the key enzymes for ABA degradation, did not show variance or even lower than that in non-primed rice seeds (data not shown). This might because that the ABA degradation process would be enhanced under high ABA concentration via a feedback inhibition mechanism, which increased the expression level of *OsABA8ox1.* However, this hypothesis needs to be examined in further studies.

The expression of *OsRamy3D* and *OsRamy3E* are regulated by the soluble sugar levels in plant tissues (Hwang et al. 1999; Loreti et al. 2003). When the seeds were germinated under stress conditions, the transcription level of *OsRamy1A* was generally low, while *OsRamy3D* and *OsRamy3E* were highly induced in response to the sugar signals, which ensured the synthesis of α-amylase in seed under stress condition (Yamaguchi 1998; Toyofuku et al. 1998; Huang et al. 2000). In present study, both Se and SA priming down-regulated the expression level of *OsRamy3D* under chilling stress, this might because that seed priming improved the total soluble sugar levels in rice seeds, which might in turn depressed the transcription of *OsRamy3D.* In contrary, the *OsRamy3E* was highly expressed in primed rice seeds as compared with non-primed ones, this result was in consist with the research of Yu et al. (1996). Qian et al. (2003) reported that an unidentified sequence in the promoter sequence of *OsRamy3E* represented similar functions to that of GAREs, indicating that *OsRamy3E* can be responded to the GA signals with the absence of GAREs. Moreover, Sheu et al. (1996) documented that the threshold of soluble sugar content to inhibit *OsRamy3E* expression was relatively high, suggesting that the increased soluble sugar contents in primed rice seeds did not reach the critical value to inhibit *OsRamy3E* expression. In addition, the research of Cheng et al. (2015) observed a synergistic effect between *OsRamy3E* and *OsRamy3B*, implying that the enhanced *OsRamy3B* expression in primed rice seeds might in turn promoted the expression of *OsRamy3E.* Nevertheless, the mechanism underlying how seed priming regulate the expression of *OsRamy3E* remained to be unknown and needs to be addressed in the future.

In addition to starch degradation, several metabolic events were also reported to contribute to stronger chilling tolerance in primed seed. Our previous research found that the better germination ability in primed rice seed under chilling stress was associated with higher respiration rate (Wang et al. 2016a; b), and such positive effect was attributed to the improved ATP levels, glycolysis metabolism and the repair and biogenesis of mitochondria in primed rice seeds (Nie et al. 2020). Besides, the primed rice seeds obsessed lower MDA content and higher level of antioxidant capacity under abiotic stresses (Zheng et al. 2016; Hussain et al. 2016). Which contribute to better membrane integrity and growth performance. Nevertheless, the crosstalk between these metabolic events and starch metabolism under chilling stress remained to be unknown, and the molecular and physiological network underlying the positive effect of seed priming against chilling stress needs to be unravel in future studies.

In present study, the selection of Se and SA priming treatments was based on the results from a series of preliminary experiments, in which the seed germination and seedling growth attributes in Se and SA primed rice seeds/seedlings were better than that of other priming treatments under chilling stress. Our reults indicated that the priming agent salicylic acid (SA) and sodium selenite (Se) may be effective in enhancing chilling tolerance of rice during germination. Selenium is reported to act as a signal molecular in plant to activate plant response to stresses and to modulate the balance between ROS production and scavenging (Kishor and Sreenivasulu 2014; Gupta and Gupta 2016). While salicylic acid is a plant hormone mainly associated with the induction of defense mechanism in plants. Ana et al. (2009) reported that SA interacted with GAs to affect plant early responses to stress. Pál et al. (2013) concluded that SA protects the plant from chilling stress by modifying the antioxidant capacity and changing expression rates of certain genes. It can be speculated that in present study, the changes of GA/ABA contents and starch degradation ability in primed rice seeds might also be attributed to the signaling effect of Se and SA. However, the possible correlations between Se and SA, and hormonal and starch metabolism in response to chilling stress were not established in present study and should be explored in the future.

## Conclusion

Both Se and SA priming significantly increased the seed germination and seedling growth attribute, and enhanced the starch degradation ability by increasing α-amylase activity and total soluble sugar content under chilling stress. Meanwhile, seed priming increased the transcription level of *OsRamy1A*, *OsRamy3B* that regulated by GA, and increased the transcription level of *OsRamy3E* that regulated by sugar signals. Furthermore, seed priming significantly improved the GA_3_ contents in rice seeds by up-regulating the expression of *OsGA3ox1, OsGA20ox1* and decreased the ABA content and the expression of *OsNCED1,* indicating that the improved starch degradation ability in primed rice seeds under chilling stress might be attributed to the increased GA_3_ and decreased ABA levels in primed rice seeds, which induced the expression of GA-mediated α-amylase. However, studies to explore how seed priming mediate hormonal metabolism and the expression of *OsRamy3E* are desperately needed.

## Supplementary Information

**Additional file 1**. **Table S1.** Sequences of primers used for RT-qPCR.

## Data Availability

The datasets used and/or analysed during the current study are available from the corresponding author on reasonable request.

## Abbreviations

ABA: Abscisic acid
CK: No priming-chilling stress
DAS: Days after sowing
EZ18: Ezao18
GA: Gibberellin acid;
GAREs: GA-responsive cis-acting elements
LY287: Liangyou287
Se: Selenium priming
SA: Salicylic priming
NT: No priming-normal temperature control
