# Supplementary material for: Enhancement in Seed Priming-Induced Starch Degradation of Rice Seed Under Chilling Stress via GA-Mediated α-Amylase Expression
Source: Rice (N Y). 2022 Mar 27;15:19. doi: 10.1186/s12284-022-00567-3 (PMC8960536; doi:10.1186/s12284-022-00567-3)
Supplement: Supplementary file 1 — Additional file 1. Table S1. Sequences of primers used for RT-qPCR. [file 12284_2022_567_MOESM1_ESM.docx]

**Table S1 Sequences of primers used for RT-qPCR**

| Gene |  | Sequence of the primers(5'-3') |
| --- | --- | --- |
| *Actin* | Forward | ATGAAGATCAAGGTGGTCGC |
|  | Reverse | GTACTCAGCCTTGGCAATCC |
| *OsRamy1A* | Forward | AGCAAGCTGAAAATCCTTGCTGCTGA |
|  | Reverse | TAATTGTTGCCGTGAGCAACGACATG |
| *OsRamy3B* | Forward | GTAGGCAGGCTCTCTAGCCTCTAGG |
|  | Reverse | GATTTTTTACTGCATCCTGAACCTG |
| *OsRamy3D* | Forward | AGGAAGGCCTCAGGGTTCCTGCCGGT |
|  | Reverse | TCTCGCAGCAAAATTGCATGAT |
| *OsRamy3E* | Forward | CATCTCCGAAGTGTGTCTGC |
|  | Reverse | ACTCCCAGTTGAATCCCTGAA |
| *OsGa3ox1* | Forward | CGGACTCGGGCTTCTTCACCT |
|  | Reverse | CGAGGAAGTAGCCGAGCGAGAC |
| *OsGa20ox1* | Forward | CCGTGGAAGGAGACGCTGTC |
|  | Reverse | GCGGCTCATCTCGTGGCAGT |
| *OsNCED1* | Forward | TCATTCCAAAACACCTTCCA |
|  | Reverse | TCCGGGGACCTCCTATGTAT |
|  |  |  |
